# Associations between persistent symptoms after mild COVID‐19 and long‐term health status, quality of life, and psychological distress

**DOI:** 10.1111/irv.12980

**Published:** 2022-03-28

**Authors:** Jin H. Han, Kelsey N. Womack, Mark W. Tenforde, D. Clark Files, Kevin W. Gibbs, Nathan I. Shapiro, Matthew E. Prekker, Heidi L. Erickson, Jay S. Steingrub, Nida Qadir, Akram Khan, Catherine L. Hough, Nicholas J. Johnson, E. Wesley Ely, Todd W. Rice, Jonathan D. Casey, Christopher J. Lindsell, Michelle N. Gong, Vasisht Srinivasan, Nathaniel M. Lewis, Manish M. Patel, Wesley H. Self, for the Influenza and Other Viruses in the Acutely Ill (IVY) Network

**Affiliations:** ^1^ Vanderbilt University Medical Center Nashville Tennessee USA; ^2^ Geriatric Research, Education, and Clinical Center Tennessee Valley Healthcare System Nashville Tennessee USA; ^3^ CDC COVID‐19 Response Team Centers for Disease Control and Prevention Atlanta Georgia USA; ^4^ Wake Forest University Baptist Medical Center Winston‐Salem North Carolina USA; ^5^ Beth Israel Deaconess Medical Center Boston Massachusetts USA; ^6^ Hennepin County Medical Center Minneapolis Minnesota USA; ^7^ Baystate Medical Center Springfield Massachusetts USA; ^8^ UCLA Medical Center Los Angeles California USA; ^9^ Oregon Health & Sciences University Portland Oregon USA; ^10^ University of Washington Seattle Washington USA; ^11^ Montefiore Medical Center The Bronx New York USA

**Keywords:** COVID‐19, COVID‐19 outcomes, long‐COVID, post‐acute sequalae of COVID‐19 (PASC), post‐COVID conditions

## Abstract

**Background:**

We sought to assess whether persistent COVID‐19 symptoms beyond 6 months (Long‐COVID) among patients with mild COVID‐19 is associated with poorer health status, quality of life, and psychological distress.

**Methods:**

This was a multicenter prospective cohort study that included adult outpatients with acute COVID‐19 from eight sites during 2‐week sampling periods from April 1 and July 28, 2020. Participants were contacted 6–11 months after their first positive SARS‐CoV‐2 to complete a survey, which collected information on the severity of eight COVID‐19 symptoms using a 4‐point scale ranging from 0 (*not present*) to 3 (*severe*) at 1 month before COVID‐19 (pre‐illness) and at follow‐up; the difference for each was calculated as an attributable persistent symptom severity score. A total attributable persistent COVID‐19 symptom burden score was calculated by summing the attributable persistent severity scores for all eight symptoms. Outcomes measured at long‐term follow‐up comprised overall health status (EuroQol visual analogue scale), quality of life (EQ‐5D‐5L), and psychological distress (Patient Health Questionnaire‐4). The association between the total attributable persistent COVID‐19 burden score and each outcome was analyzed using multivariable proportional odds regression.

**Results:**

Of the 2092 outpatients with COVID‐19, 436 (21%) responded to the survey. The median (IQR) attributable persistent COVID‐19 symptom burden score was 2 (0, 4); higher scores were associated with lower overall health status (aOR 0.63; 95% CI: 0.57–0.69), lower quality of life (aOR: 0.65; 95%CI: 0.59–0.72), and higher psychological distress (aOR: 1.40; 95%CI, 1.28–1.54) after adjusting for age, race, ethnicity, education, and income.

**Conclusions:**

In participants with mild acute COVID‐19, the burden of persistent symptoms was significantly associated with poorer long‐term health status, poorer quality of life, and psychological distress.

## 1 INTRODUCTION

Through November 2021, over 260 million people had been diagnosed with coronavirus disease 2019 (COVID‐19) worldwide.^1^ A large body of research suggests that a substantial proportion of people with acute COVID‐19 develop post‐acute sequalae of COVID‐19 (PASC),^2^ or Long‐COVID, characterized by symptoms that persist for months after the initial infection.^3^, ^4^, ^5^, ^6^, ^7^ Most prior studies evaluating PASC were conducted among patients who were hospitalized with moderate to severe COVID‐19, which may not be generalizable to patients with mild COVID‐19 who were never hospitalized. Furthermore, most prior studies did not account for the presence of symptoms before a diagnosis of COVID‐19 illness, making it difficult to understand if persistent post‐illness symptoms represented a return to a pre‐illness baseline or new morbidity attributable to COVID‐19.

A growing body of literature also suggests that many people report poor quality of life and high psychological distress months after an initial diagnosis of COVID‐19.^4^, ^5^, ^6^, ^8^ The mechanisms and risk factors for long‐term morbidity following COVID‐19 illness remain poorly understood. To address these knowledge gaps, we sought to characterize COVID‐19 symptom trajectories before, during, and 6–11 months after acute mild COVID‐19 illness and evaluate long‐term self‐reported health status, quality of life, and psychological distress. We hypothesized that: (1) some outpatients with mild COVID‐19 would exhibit substantial, persistent symptoms at 6–11 months after initial diagnosis; and (2) the burden of persistent symptoms would be associated with lower health status and quality of life, and higher psychological distress.

## 2 METHODS

### 2.1 Study design, setting, and participants

A multicenter prospective cohort study conducted by the Influenza and Other Viruses in the Acutely Ill (IVY) Network, which is a collaboration among 21 academic medical centers in the United States (US) and the Centers for Disease Control and Prevention (CDC) and coordinated at Vanderbilt University Medical Center.^9^, ^10^ As previously described,^11^, ^12^, ^13^ sites obtained lists of outpatient adults who tested positive for SARS‐CoV‐2 by reverse transcription polymerase chain reaction (RT‐PCR) testing of a nasal swab during discrete two‐week periods between April 1, 2020, and July 28, 2020.

Eight IVY centers from seven US states participated in this analysis. This analysis included participants who indicated that they had symptoms of COVID‐19 at the time of the positive SARS‐CoV‐2 reverse transcriptase‐polymerase chain reaction (RT‐PCR) test and were treated as outpatients. Outpatient status was defined as SARS‐CoV‐2 testing conducted at a clinic, ambulatory testing center, or emergency department without subsequent hospitalization.

This analysis was determined to be a public health surveillance activity with a waiver of informed consent by participating sites and CDC and was conducted in compliance with CDC policies and applicable federal law (45 C.F.R. part 46.102(l)(2), 21 C.F.R. part 56; 42 U.S.C. Sect. 241(d); 5 U.S.C. Sect. 552a; 44 U.S.C. Sect. 3501 et seq).

### 2.2 Data collection

Data were collected through two sources: the local sites' electronic medical records and by Twilio (Cloud communications platform, San Francisco, CA). The electronic medical record was used as a source for SARS‐CoV‐2 test dates and results. Twilio is a software application that sent survey links to participants via SMS text message on February 4, 2021, with serial text message reminders sent to those who had not responded over the subsequent month. The survey assessed symptoms at 6–11 months after the initial positive SARS‐CoV‐2 test well as self‐assessed health status, quality of life, and psychological distress. For this project, Twilio was integrated into a REDCap data collection tool, which was used to store all data.

### 2.3 Symptom ascertainment by Twilio survey

The survey, available in the supporting information, was administered in two parts. Part 1 of the survey asked participants if they were still experiencing physical or mental problems related to COVID‐19. Those who had persistent symptoms at long‐term follow‐up were asked to report the most severe symptom still present. Those who had no persistent symptoms at long‐term follow‐up were asked how long it took to recover from their COVID‐19 symptoms.

All participants who completed Part 1 were also invited to complete Part 2 of the survey, which collected demographic data and asked participants to classify the severity of eight symptoms associated with PASC in previous studies (fatigue, difficulty sleeping, difficulty concentrating, difficulty completing usual daily tasks, shortness of breath, loss of taste, loss of smell, and hair loss) at three time points: 1 month before the onset of COVID‐19 symptoms (pre‐illness baseline), at the worst point during acute COVID‐19 (peak symptoms), and currently at the time of survey completion (long‐term follow‐up, 6–11 months after the initial positive SARS‐CoV‐2 test). The severity of each symptom was classified using a 4‐level ordinal scale that ranged from 0 (*not present*) to 3 (*severe*) (Table S1).

An attributable persistent COVID‐19 symptom severity score was calculated for each individual symptom as: long‐term follow‐up score minus pre‐illness baseline score. For example, if a patient reported a fatigue score of 3 at long‐term follow‐up and a baseline fatigue score of 1, the persistent COVID‐19 fatigue score would be 2. An attributable persistent score ≥1 indicated symptoms were more severe 6–11 months after acute COVID‐19 than before COVID‐19. The term “attributable” was used to indicate that the score accounted for pre‐illness baseline symptoms. The term “persistent” was used to indicate the symptoms were present beyond 6 months from the onset of COVID‐19. A total attributable persistent COVID‐19 symptom burden score was calculated by summing the eight symptom‐specific scores. This resulted in a total score with a range of −24 to 24, with positive scores indicating symptoms beyond a pre‐COVID baseline that were still present at follow‐up.

### 2.4 Outcome ascertainment by Twilio survey

Part 2 of the Twilio survey also asked participants to report information on three outcomes: overall health status, quality of life, and psychological distress. For overall health status, participants rated their self‐assessed health using the EuroQol Visual Analog Scale (EQ‐VAS) for 1 month before the COVID‐19 illness (pre‐illness baseline) and at long‐term follow‐up. The EQ‐VAS ranges from 0 (worst health imaginable) to 100 (best health imaginable).^14^ A change in EQ‐VAS by ≥8 points between the pre‐illness baseline and long‐term follow‐up was considered clinically important.^15^, ^16^

Participants reported quality of life at long‐term follow‐up using the EQ‐5D‐5L, a 5‐question instrument that captures impairments in mobility, self‐care, usual activities, pain/discomfort, and anxiety/depression.^17^ EQ‐5D‐5L scores were summarized using the index value validated for the US population.^18^ An index value of 1.0 indicated perfect health, a value of 0.0 indicated death, and a value <0.0 indicated quality of life the participant rated as worse than death.^18^

Participants reported information on psychological distress at long‐term follow‐up using the Patient Health Questionnaire‐4 (PHQ‐4).^19^ This 4‐item survey was used to grade the severity of depression and anxiety symptoms, with scores ranging from 0 (*no symptoms*) to 12 (*severe symptoms*). A score of ≤2 was interpreted as no psychological distress.

### 2.5 Data analysis

COVID‐19 symptom trajectory was summarized by reporting median and interquartile range (IQR) values and displaying box‐and‐whisker plots for symptom severity scores at three time points: pre‐illness baseline, peak acute COVID‐19, and long‐term follow‐up (6–11 months after the initial positive SARS‐CoV‐2 test). The association between the total attributable persistent COVID‐19 symptom burden score and each outcome (overall health status, quality of life, and psychological distress) was evaluated using multivariable proportional odds regression models. Proportional odds regression was used due to the non‐parametric distribution of the outcome variables. In each model, the total attributable persistent COVID‐19 symptom burden score was the primary independent variable, the outcome score was the dependent variable, and covariables included age, race, ethnicity, education level, income level, and pre‐COVID overall health status (baseline EQ‐VAS score). Details of the model covariables are described in Table S2. The proportional odds regression models produced adjusted odds ratios (aOR) with 95% confidence intervals (95% CI) summarizing the association between the total attributable persistent COVID‐19 symptom burden score and each of the three outcomes. An aOR < 1 indicated an association between higher persistent symptom burden and lower overall health status and quality of life (worse outcomes). An aOR > 1 indicated an association between higher persistent symptom burden and higher psychological distress (worse outcome). Analyses were conducted with SAS Version 9.4 (SAS Institute, Cary, NC).

## 3 RESULTS

### 3.1 Part 1 survey results

**FIGURE 1:**
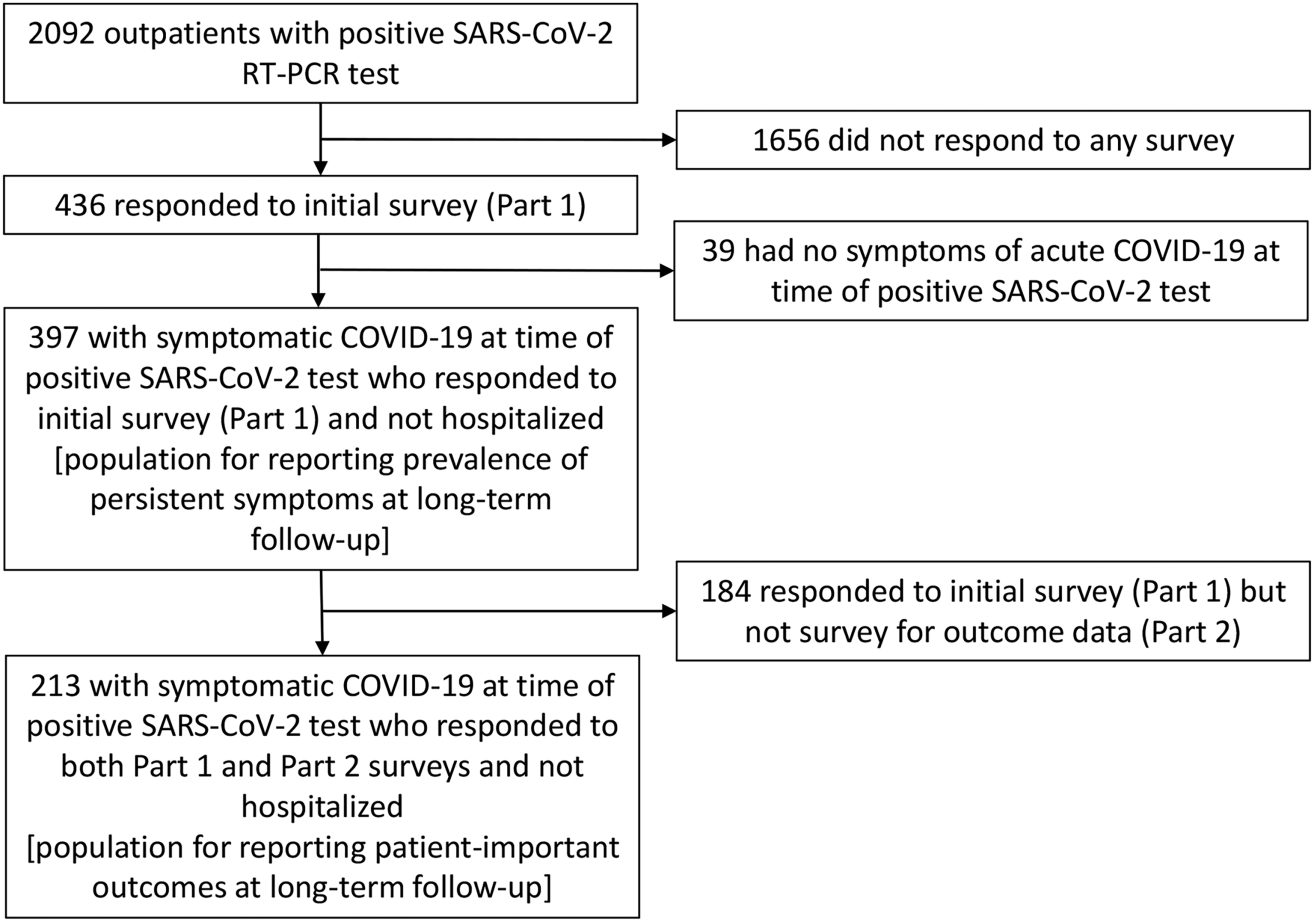
Flow diagram of patient participation. RT‐PCR, reverse transcription polymerase chain reaction

During the two‐week sampling periods between April 1 and July 28, 2020, eight participating sites submitted a total of 2092 outpatients who tested positive for SARS‐CoV‐2, and of these, 436 (21%) responded to Part 1 of the survey (Figure 1). Of the 436 respondents, 39 were excluded because they did not have any symptoms of COVID‐19 at the time of the positive SARS‐CoV‐2 test resulting in an analytic sample of 397 participants.

Among 397 participants, 346 (87%) respondents took the survey in English and 51 (13%) respondents in Spanish. The median time between the positive SARS‐CoV‐2 test and long‐term follow‐up survey completion was 8 months (range: 6–11 months). Among 397 participants, 176 (44%) had persistent COVID‐19 symptoms at the time of long‐term follow‐up, and 221 (56%) had no persistent symptoms. Among the 176 who had persistent symptoms and completed Part 1, the symptoms most frequently reported as the most severe among all eight potential persistent symptoms were fatigue (31%), shortness of breath (20%), difficulty with concentration (9%), and loss of smell (9%) (Table S3). Of 221 participants who did not have persistent symptoms (i.e., were recovered) at long‐term follow‐up, 122 (55%) had recovered in <1 month, 74 (33%) recovered in 1–3 months, 20 (9%) had recovered in 3–6 months, and 5 (2%) had recovered after 6 months.

### 3.2 Part 2 survey results

Part 2 of the survey with outcome data was completed by 213 participants; 105/176 (60%) participants who reported persistent symptoms on the Part 1 survey went on to complete Part 2, and 108/221 (49%) who did not report persistent symptoms in Part 1 also completed Part 2. The median (IQR) age of these participants was 45 (33, 57) years, 141 (66%) were women, 43 (20%) were Hispanic, 32 (15%) were non‐Hispanic Black, 40 (19%) had a high school degree or below, and 56 (26%) had a household income less than $50 000 (Table 1). The median (IQR) pre‐Illness EQ‐VAS overall health status score was 86 (75, 93).

**TABLE 1:** Adult outpatients with acute COVID‐19 (*n* = 213 of 397 participants who responded to Part 2 of the long‐term outcome survey)

Demographics	N = 213
Age in years, median (IQR)	45 (33, 57)
Female Sex, no. (%)	141 (66.2%)
Hispanic Ethnicity, no. (%)	43 (20.2%)
Non‐Hispanic Black, no. (%)	32 (15.0%)
Race, no. (%)	
Black	33 (15.5%)
American Indian or Alaska Native	1 (0.5%)
Asian	4 (1.9%)
Native Hawaiian or Other Pacific Islander	1 (0.5%)
White	146 (68.5%)
Other	26 (12.2%)
Prefer not to answer	2 (0.9%)
Education level, no. (%)	
Grades 9–11 (Some high school)	7 (3.3%)
Grade 12 or GED (High school graduate)	33 (15.5%)
College 1–3 years (Some college or technical school)	54 (25.4%)
College 4 years or more (College graduate)	116 (54.5%)
Prefer not to answer	3 (1.4%)
Income	
Less than $25,000	17 (8.0%)
$25,000–$34,000	19 (8.9%)
$35,000–$49,000	20 (9.4%)
$50,000–$74,000	33 (15.5%)
$75,000 or more	100 (46.9%)
Prefer not to answer/Do not know	24 (11.3%)
Pre‐illness overall health status score on EuroQol Visual Analogue Scale, median (IQR)	86 (75, 93)

Abbreviations: IQR = interquartile range, GED = General Educational Development.

**FIGURE 2:**
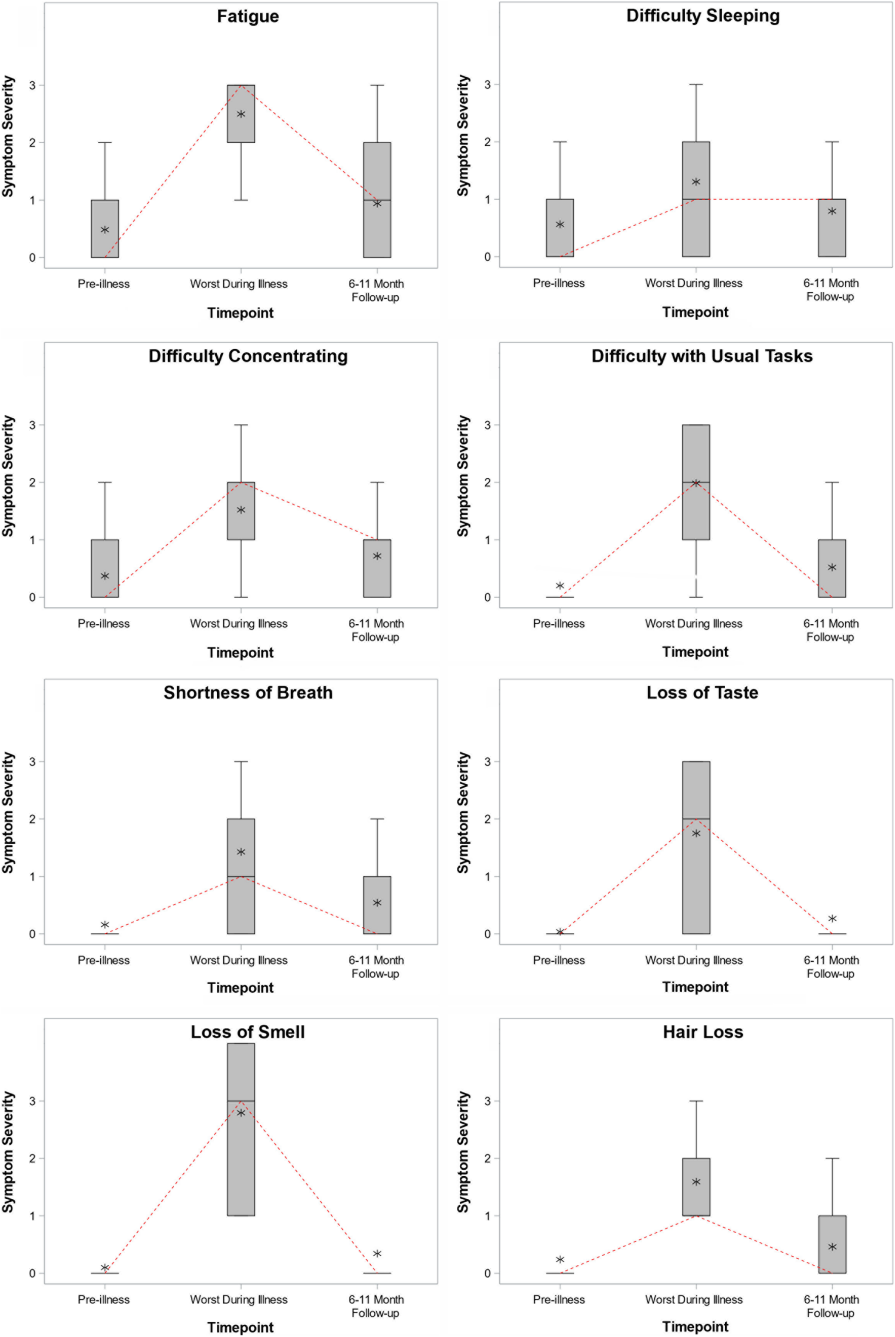
Symptom trajectories from 1 month before COVID‐19 (pre‐illness baseline), during peak acute COVID‐19 (worst during illness), and at long‐term follow‐up (6–11 months after the positive SARS‐CoV‐2 test) for 8 COVID‐19 symptoms. Patients rated their symptoms using a 4‐level Likert scale ranging from 0 (*no symptoms*) to 3 (*severe symptoms*). At each time point, a summary of symptom severity among all participants is plotted with a box plot, with the median score represented by the horizontal line, the interquartile range (25th percentile to 75th percentile) represented by the box, the full range represented by the outer brackets, and the mean represented by the star. A dashed line connects the median from each time point to highlight changes in the central tendency over time

COVID‐19 symptom trajectories from 1 month before COVID‐19 symptom onset (pre‐illness), during peak acute COVID‐19, and to long‐term follow‐up (6 to 11 months after the initial positive SARS‐CoV‐2 test) are displayed in Figure 2. Several patterns of symptom trajectories were observed. Loss of taste and smell were very rare before COVID‐19, tended to become severe for many participants during acute COVID‐19, and then completely resolved for nearly all participants by long‐term follow‐up. Shortness of breath and difficulty with usual tasks were rare before COVID‐19, became severe for many participants during peak COVID‐19, and persisted for a minority at long‐term follow‐up. Fatigue and difficulty concentrating were present at low levels for many participants prior to COVID‐19, peaked at high levels during acute illness, and then tended to decline somewhat by long‐term follow‐up, but not all the way down to pre‐illness levels.

Among 213 respondents to Part 2 of the survey, 154 (72%) had at least one symptom that was more severe at long‐term follow‐up than pre‐illness baseline (attributable persistent symptom score ≥1). The percentage of patients with an attributable persistent symptom score ≥1 ranged across symptoms from 17% for hair loss to 41% for fatigue (Table 2). Of note, an attributable persistent symptom score ≥1 was reported by 33% for difficulty concentrating and 32% for shortness of breath. Furthermore, 65 (31%) respondents had at least one symptom with an attributable persistent symptom score ≥2. The percentage of patients with an attributable persistent symptom score ≥2 ranged from 4% for sleep to 11% for fatigue.

**TABLE 2:** Attributable persistent COVID‐19 symptom scores for eight COVID‐19 symptoms^a^

COVID‐19 symptom	Attributable persistent COVID‐19 symptom severity score, median (IQR)	Attributable persistent COVID‐19 symptom severity score ≥1, no. (%) of patients	Attributable persistent COVID‐19 symptom severity score ≥2, no. (%) of patients
Fatigue	0 (0, 1)	87 (40.8%)	24 (11.3%)
Difficulty sleeping	0 (0, 0)	51 (23.9%)	8 (3.8%)
Difficulty with concentration	0 (0, 1)	70 (32.9%)	11 (5.2%)
Difficulty performing usual tasks	0 (0, 1)	60 (28.2%)	15 (7.0%)
Shortness of breath	0 (0, 1)	67 (31.5%)	15 (7.0%)
Loss of taste	0 (0, 0)	38 (17.8%)	11 (5.2%)
Loss of smell	0 (0, 0)	45 (21.1%)	13 (6.1%)
Hair loss	0 (0, 0)	37 (17.4%)	13 (6.1%)
Total	2 (0, 4)	154 (72.3%)	65 (30.5%)

Abbreviations: IQR = interquartile range.

^a^ Participants were asked to rate the severity of eight symptoms at three time points: before COVID‐19 (pre‐illness baseline), during the peak of their COVID‐19 illness (peak symptoms), and at 6–11 months after the positive SARS‐CoV‐2 test (long‐term follow‐up). The severity of symptoms was rated using a 4‐level Likert scale ranging from 0 (*no symptoms*) to 3 (*severe symptoms*). The attributable persistent COVID‐19 score was calculated as the long‐term follow‐up score minus the pre‐illness baseline score. This table includes 213 participants who responded to Part 2 of the long‐term follow‐up survey.

### 3.3 Total attributable persistent COVID‐19 symptom burden scores and association with outcomes

The total attributable persistent COVID‐19 symptom burden score was calculated for each participant by summing the attributable persistent symptom score for each of the eight symptoms. Among the 213 respondents to Part 2 of the survey, the median (IQR) total attributable persistent COVID‐19 symptom burden score was 2 (0, 4); 144 (68%) respondents had a score ≥1, indicating a greater burden of symptoms at long‐term follow‐up compared with before the onset of COVID‐19 symptoms.

At long‐term follow‐up, the median (IQR) score for EQ‐VAS was 78 (69, 89), for EQ‐5D‐5L was 0.90 (0.79, 1.00), and for PHQ‐4 was 2 (0, 4) (Table 3). The median (IQR) decline in EQ‐VAS score between pre‐illness baseline and long‐term follow‐up was 5 (0, 15) points, with 86 (40%) participants having a decline of ≥8 points, which was considered clinically important.

**TABLE 3:** Participant‐reported outcomes on the long‐term outcome survey completed 6–11 months after a positive test for SARS‐CoV‐2^a^

Outcome^b^	Outcome score at long‐term follow‐up^c^, median (IQR)	Association between the total attributable persistent COVID‐19 symptom burden score and outcome, adjusted odds ratio (95% CI)
Overall health status measured by EQ‐VAS	78 (60, 89)	0.63 (0.57–0.69)
Quality of life measured by EQ‐5D‐5L	0.90 (0.79, 1.00)	0.65 (0.59–0.72)
Psychological stress measured by PHQ‐4	2 (0, 4)	1.40 (1.28–1.54)

Abbreviations: IQR = interquartile range, EQ‐VAS = EuroQol Visual Analog Scale;  PHQ‐4 = Patient Health Questionnaire‐4.

^a^ The association between the total attributable persistent COVID‐19 burden score (a measure of burden of persistent COVID‐19 symptoms) and each outcome was analyzed using multivariable proportional odds regression. This table includes 213 participants who responded to Part 2 of the long‐term follow‐up survey.

^b^ Overall health status was measured using the EuroQol visual analogue scale (EQ‐VAS), which asked participants to rate their health from 0 (worst health) to 100 (best health). Quality of life was measured with the EQ‐5D‐5L, which measures the participant's mobility, self‐care, activities, pain, and anxiety, and is summarized with a utility score ranging from <0 (worse than death) to 1.0 (perfect health). Psychological distress was measured with the Patient Health Questionnaire‐4 (PHQ‐4), a 4‐item questionnaire that grades the severity of depression and anxiety and ranges from 0 (no symptoms) to 12 (severe psychological distress).

^c^ Long‐term follow‐up was 6–11 months after the first positive SARS‐CoV‐2 test.

The total attributable persistent COVID‐19 burden score was significantly associated with worse outcomes at long‐term follow‐up, including lower overall health status measured by EQ‐VAS (aOR 0.63; 95% CI: 0.57–0.69), lower quality of life measured by the EQ‐5D‐5L (aOR: 0.65; 95%CI: 0.59–0.72), and more psychological distress measured by PHQ‐4 (aOR: 1.40; 95%CI, 1.28–1.54).

## 4 DISCUSSION

PASC, or Long‐COVID, is characterized by COVID‐19 symptoms that persist after the acute illness, and are well‐documented among patients who had moderate‐to‐severe COVID‐19 treated in a hospital or intensive care unit.^3^, ^4^, ^5^, ^6^, ^7^ Our study adds to this literature by showing that approximately 44% of outpatients with COVID‐19 who responded to a Twilio survey had persistent symptoms 6–11 months after an initial positive SARS‐CoV‐2 test. Among the most common persistent symptoms were fatigue, shortness of breath, and difficulty concentrating. Importantly, we observed that the severity of persistent symptoms was strongly associated with poorer long‐term patient‐important outcomes, including overall health status, quality of life, and psychological distress. These results suggest that prevention of SARS‐CoV‐2 infection through vaccination or other measures, and consequent prevention of attendant post‐COVID morbidity, may be an important avenue to improve long‐term health and quality of life for the millions of people who have had COVID‐19 and those who are likely to get it in the future.

A recent meta‐analysis of 15 studies, focused mostly on patients who were hospitalized for acute COVID‐19, reported that 80% of patients had one or more long‐term symptoms, including 58% with fatigue, 27% with attention/concentration difficulties, 25% with hair loss, and 24% with shortness of breath at up to 110 days after infection.^7^ Few prior studies have evaluated persistent symptoms among outpatients with mild COVID‐19. In patients with mild COVID‐19 illness, persistent symptoms have been reported in 15% to 33% of cases with fatigue, shortness of breath, and loss of taste and smell being the most common residual symptoms.^3^, ^20^ Graham *et al* ^21^ studied 50 outpatients with COVID‐19 and found comparatively higher prevalence of persistent symptoms at 6 months, including 84% with fatigue, 64% with loss of taste, 74% with loss of smell, 40% with depression and/or anxiety, and 38% with shortness of breath. The prevalence of persistent symptoms observed in our current study was between the observations previously reported and demonstrated similar patterns, with fatigue the most common persistent symptom and substantial numbers of patients with persistent difficulties with thinking and breathing.

“Brain fog” following acute COVID‐19 has attracted considerable interest and is a well‐documented complication following severe COVID‐19 treated in the hospital.^3^, ^22^, ^23^ While the cognitive domains affected by “brain fog” after COVID‐19 are not well characterized, attention and working memory are likely involved.^24^ We observed that 32% of our outpatient cohort still had not returned to their pre‐illness concentration status 6–11 months after acute illness. Other outpatient COVID‐19 cohorts have reported prevalences of persistent “brain fog” ranging from 2% to 81%.^3^, ^21^ Future studies using formal neuropsychological testing are needed to fully characterize the cognitive deficits underlying this commonly reported “brain fog” symptom to facilitate greater mechanistic understanding and help develop potential treatments.

A major strength of this study was the analysis showing an association between persistent COVID‐19 symptoms and patient‐centered outcomes (overall health status, quality of life, and psychological distress). Long‐term morbidity reported among people who had mild COVID‐19 in this study was lower than that reported by patients who were hospitalized for severe COVID‐19 in other studies.^3^, ^4^, ^5^, ^6^, ^7^ Therefore, patients with milder acute disease may be at lower risk for severe long‐term morbidity than those with more severe illness during the acute phase of COVID‐19.^4^, ^5^ Nonetheless, impaired long‐term health, reduced quality of life, and increased psychological distress post‐COVID were reported by a substantial number of patients treated as an outpatient for COVID‐19 in this study, and the burden and severity of persistent symptoms correlated with long‐term health. Therefore, people who are treated as outpatients for mild COVID‐19, but have a high burden of persistent symptoms, may benefit from access to the Post‐COVID Recovery Clinics that have been developed for inpatients and ICU survivors.^25^, ^26^, ^27^

One novel aspect of our study was the implementation of automated survey delivery using Twilio; survey links were sent to subjects' cellular phones using SMS text messaging and automatically sent reminders over a one‐month period. We achieved a 21% overall response rate without providing advanced notice to the subject that an online survey would be sent and without providing financial reimbursement to responders. Obtaining long‐term follow‐up is often labor intensive and costly, especially for multi‐center studies. Considerable effort is often expended in trying to contact patients for follow‐up using traditional techniques, such as telephone banks. Using automated survey delivery tools could increase the feasibility and decrease the cost of obtaining long‐term follow‐up while also allowing participants to complete surveys at their own convenience. A limitation of this approach is the low response rate compared with traditional methods. Response rates could be improved by providing subject reimbursement, notifying subjects to expect an SMS text message with an online survey, or following up patients who do not respond to text‐based surveys with telephone calls.^28^

Our study had some additional limitations. First, the study population was a convenience sample of people who had COVID‐19 as an outpatient in the United States during the spring and summer of 2020 and completed a cellular phone‐based survey. The study was subject to response bias, as people with persistent symptoms may have been more likely to answer the survey than those without persistent symptoms. Conversely, patients who were substantially debilitated at the time of the survey may have been less likely to complete the survey if they were not able to answer cellular phone messages. Second, answering the survey required access to a cellular phone and to be able to communicate in English or Spanish. This systematically excluded patients who do not use a cellular phone, estimated at 3% of the US population,^29^ and those who do not speak English or Spanish. Third, a standardized approach for quantifying COVID‐19 symptoms has not been established. The attributable persistent symptom score may not fully capture differences in the health burden carried by different types of symptoms and cannot account for differences in each patient's interpretation (e.g., a score of 2 vs. 3) of the severity scoring system. Alternative strategies for quantifying COVID‐19 symptoms may emerge in the future. Fourth, participants were contacted at 6–11 months after acute COVID‐19 and asked to recall symptoms they had prior to COVID‐19 and during peak acute COVID‐19; this method of collecting information on symptoms from earlier time periods may be subject to inaccuracies and recall bias. Fifth, and relatedly, the exclusion of patients with no COVID‐19 symptoms may also lead to overestimation of the burden of persistent symptoms within the broader patient population. Sixth, while the statistical models evaluating the association between persistent symptoms and long‐term outcomes were adjusted for some potential confounders, residual confounding is possible. Seventh, our study was conducted early in the pandemic, and the findings may not be generalizable to the effect of subsequent waves, with other variants of SARS‐CoV‐2, such as Delta or Omicron. Lastly, we did not include a control group of patients without COVID‐19. Therefore, it is uncertain if our findings are unique to COVID‐19 or are observed in other respiratory infections. However, Taquet *et al* reported that patients with COVID‐19 were more likely to have neurological and psychological sequelae compared with influenza and other non‐SARS‐CoV‐2 respiratory infections.^30^

## 5 CONCLUSIONS

Among a group of patients with mild COVID‐19 not requiring hospitalization who participated in this cohort study, almost half had persistent symptoms after 6–11 months. Although long‐term morbidity rates reported here were lower than those in cohorts of hospitalized patients, persistent symptoms were significantly associated with poorer long‐term health status, poorer quality of life, and psychological distress. Medical practitioners should be aware of the potential for long‐term symptoms even among mild COVID‐19 cases and provide treatment options (e.g., specialized care) to patients accordingly.

## AUTHOR CONTRIBUTIONS

**Kelsey Womack:** Conceptualization; data curation; investigation; methodology; project administration; resources; software; supervision. **Mark W. Tenforde:** Funding acquisition; investigation; resources. **D. Clarke Files:** Data curation; investigation. **Kevin W. Gibbs:** Data curation; investigation. **Nathan I. Shapiro:** Data curation; investigation. **Matthew E. Prekker:** Data curation; investigation. **Heidi L. Erickson:** Data curation; investigation. **Jay S. Steingrub:** Data curation; investigation. **Nida Qadir:** Data curation; investigation. **Akram Khan:** Data curation; investigation. **Catherine L. Hough:** Conceptualization; data curation; investigation. **Nicholas J. Johnson:** Data curation; investigation. **E. Wesley Ely:** Investigation. **Todd W. Rice:** Conceptualization; data curation; investigation. **Jonathan D. Casey:** Data curation; investigation. **Christopher J. Lindsell:** Conceptualization; formal analysis; investigation; methodology; project administration; validation. **Michelle N. Gong:** Data curation; investigation. **Vasisht Srinivasan:** Data curation; investigation. **Nathaniel M. Lewis:** Conceptualization; investigation; resources. **Manish M. Patel:** Conceptualization; investigation; resources; supervision. **Wesley H. Self:** Conceptualization; funding acquisition; investigation; methodology; project administration; supervision.

### PEER REVIEW

The peer review history for this article is available at https://publons.com/publon/10.1111/irv.12980.

## Supporting information




**Table S1.** Participants were asked to rate the severity of the 8 COVID‐19 symptoms shown in this table. The severity of each symptom was classified as: 0 (none), 1 (slight), 2 (moderate), or 3 (severe). At the time of survey completion 6–11 months after the initial positive SARS‐CoV‐2 test, participants were asked to classify the severity of each symptom for three time points: one month before COVID‐19 (pre‐illness baseline), at the worst point during acute COVID‐19 (peak symptoms), and currently at the time of survey completion. The grading of symptoms for the baseline and peak timepoint relied on the memory of symptoms that were present earlier.
**Table S2**. Covariables used in multivariable regression models.
**Table S3.** The most severe persistent COVID‐19 symptom at 6 to 11 months after a positive SARS‐CoV‐2 test among 176 people with mild acute COVID‐19 treated as an outpatient. These 176 people responded to Part 1 of the Twilio survey that they had persistent symptoms at the time of survey completion.

## Data Availability

A de‐identified dataset for this study may be requested from CDC (www.cdc.gov/info).
