# Supplementary material for: Associations between persistent symptoms after mild COVID‐19 and long‐term health status, quality of life, and psychological distress
Source: Influenza Other Respir Viruses. 2022 Mar 28;16(4):680–9. doi: 10.1111/irv.12980 (PMC9111447; doi:10.1111/irv.12980)
Supplement: Supplementary file 1 — Table S1. Participants were asked to rate the severity of the 8 COVID‐19 symptoms shown in this table. The severity of each symptom was classified as: 0 (none), 1 (slight), 2 (moderate), or 3 (severe). At the time of survey completion 6–11 months after the initial positive SARS‐CoV‐2 test, participants were asked to classify the severity of each symptom for three time points: one month before COVID‐19 (pre‐illness baseline), at the worst point during acute COVID‐19 (peak symptoms), and currently at the time of survey completion. The grading of symptoms for the baseline and peak timepoint relied on the memory of symptoms that were present earlier. Table S2. Covariables used in multivariable regression models. Table S3. The most severe persistent COVID‐19 symptom at 6 to 11 months after a positive SARS‐CoV‐2 test among 176 people with mild acute COVID‐19 treated as an outpatient. These 176 people responded to Part 1 of the Twilio survey that they had persistent symptoms at the time of survey completion. [file IRV-16-680-s001.pdf]

Supplemental Appendix

Table of Contents

Acknowledgments……………………………………………………………..…………….. 2

Part 1 - Brief Survey………………………………………………………………………………. 3

Part 2 - Detailed Survey…………………………………………………………………………….. 4

Supplemental Tables ……………………………………………………………………………….. 8

Table S1. Participants were asked to rate the severity of the 8 COVID-19 symptoms shown in this table………………………………………………………………………………… 8

Table S2. Covariables used in multivariable regression models………………………….. 9

Table S3. The most severe persistent COVID-19 symptom at 6 to 11 months after a positive SARS-CoV-2 test…………………………………………………………………... 10

Acknowledgments

In addition to the authors, the following contributed to this project:

Baystate Medical Center: Lori-Ann Kozikowski, Cynthia Kardos, Lesley De Souza, Raelynn Defeo, Denise Gallant, Sarah Romain, Scott Ouellette

Hennepin County Medical Center: Audrey Hendrickson, Sean Caspers

Montefiore Medical Center: Rahul Nair, Amira Mohammed

Oregon Health & Science University: Raju Reddy, Olivia Krol, Madeline McDougal, Milad Karami Jouzestani, Kinjal Mistry, Andrea Luong

Wake Forest University Baptist Medical Center: Madeline Hicks, Leigha Landreth, Mary LaRose, Lisa Parks

University of California – Los Angeles: Adreanne Rivera, Marianne Bernardo, Trevor Frankel

University of Washington: Christine Crider, Thomas Paulsen, Kyle Steinbock, Daniel Henning

Vanderbilt University Medical Center: Adrienne Baughman, Jakea Johnson, Kim Hart

Part 1 – Brief Survey

Our records indicate that you tested positive for COVID-19 in the spring or summer of 2020. Some people don't feel sick when infected, did you have any symptoms when you tested positive?	❑ Yes<br>❑ No
Are you still experiencing any problems with your physical or mental health that you believe is related to your COVID-19 illness?	❑ Yes<br>❑ No
What is the main/worst problem you are still having?	❑ Fatigue<br>❑ Difficulty sleeping/insomnia<br>❑ Difficulty concentrating<br>❑ Difficulty with usual daily tasks (such as work activities or caring for family members)<br>❑ Shortness of breath<br>❑ Loss of taste<br>❑ Loss of smell<br>❑ Hair loss<br>❑ Anxiety<br>❑ Depression

Part 2 – Detailed Survey

**Overall Health Status (EQ-VAS)**	
We would like to know how good or bad your health was before you were ill with COVID-19. The scale is numbered from 0 to 100. 100 means the best health you can imagine. 0 means the worst health you can imagine. Tap and move the slider to the point on the scale that indicates how your health was before you were ill with COVID-19.	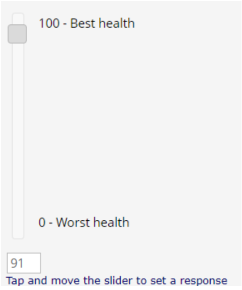
We would like to know how good or bad your health is TODAY. The scale is numbered from 0 to 100. 100 means the best health you can imagine. 0 means the worst health you can imagine. Tap and move the slider to the point on the scale that indicates how your health is TODAY.	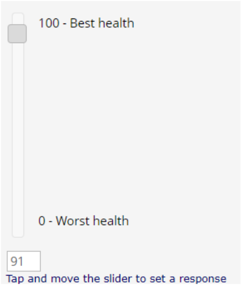
**Severity of COVID-19 Symptoms**<br>The following questions ask about symptoms you may have experienced while ill with COVID-19. Our records indicate that you had a positive test for COVID-19 in the Spring or Summer of 2020. Think back to that time before and during your illness when answering these questions. You will be asked to think about three different time frames.<br><br>First, you will be asked about how you were feeling before you were sick with COVID-19. Any symptoms you experienced during this time are expected to be unrelated to COVID-19 and represent your health prior to getting COVID-19. Second, you will be asked about your experiences when your COVID-19 illness was at its worst. Third, you will be asked about how you are feeling currently.	
One month before your illness with COVID-19, how would you rate your level of fatigue?	❑ No fatigue<br>❑ Slight fatigue<br>❑ Moderate fatigue<br>❑ Severe fatigue
When your COVID-19 illness was at its worst how would you rate your level of fatigue?	❑ No fatigue<br>❑ Slight fatigue<br>❑ Moderate fatigue<br>❑ Severe fatigue
How would you rate your current level of fatigue?	❑ No fatigue<br>❑ Slight fatigue<br>❑ Moderate fatigue<br>❑ Severe fatigue
One month before your illness with COVID-19, how would you rate your ability to sleep?	❑ No problems sleeping<br>❑ Slight problems sleeping

	❑ Moderate problems sleeping<br>❑ Severe problems sleeping
When your COVID-19 illness was at its worst how would you rate your ability to sleep?	❑ No problems sleeping<br>❑ Slight problems sleeping<br>❑ Moderate problems sleeping<br>❑ Severe problems sleeping
How would you rate your ability to sleep?	❑ No problems sleeping<br>❑ Slight problems sleeping<br>❑ Moderate problems sleeping<br>❑ Severe problems sleeping
One month before your illness with COVID-19, how would you rate your ability to concentrate?	❑ No problems concentrating<br>❑ Slight problems concentrating<br>❑ Moderate problems concentrating<br>❑ Severe problems concentrating
When your COVID-19 illness was at its worst how would you rate your ability to concentrate?	❑ No problems concentrating<br>❑ Slight problems concentrating<br>❑ Moderate problems concentrating<br>❑ Severe problems concentrating
How would you rate your ability to concentrate?	❑ No problems concentrating<br>❑ Slight problems concentrating<br>❑ Moderate problems concentrating<br>❑ Severe problems concentrating
One month before your illness with COVID-19, how would you rate your ability to complete your usual tasks?	❑ No problems completing usual tasks<br>❑ Slight problems completing usual tasks<br>❑ Moderate problems completing usual tasks<br>❑ Severe problems completing usual tasks
When your COVID-19 illness was at its worst how would you rate your ability to complete your usual tasks?	❑ No problems completing usual tasks<br>❑ Slight problems completing usual tasks<br>❑ Moderate problems completing usual tasks<br>❑ Severe problems completing usual tasks
How would you rate your ability to complete your usual tasks?	❑ No problems completing usual tasks<br>❑ Slight problems completing usual tasks<br>❑ Moderate problems completing usual tasks<br>❑ Severe problems completing usual tasks
One month before your illness with COVID-19, how would you rate your level of shortness of breath?	❑ No shortness of breath<br>❑ Slight shortness of breath<br>❑ Moderate shortness of breath<br>❑ Severe shortness of breath

When your COVID-19 illness was at its worst how would you rate your level of shortness of breath?	❑ No shortness of breath<br>❑ Slight shortness of breath<br>❑ Moderate shortness of breath<br>❑ Severe shortness of breath
How would you rate your level of shortness of breath?	❑ No shortness of breath<br>❑ Slight shortness of breath<br>❑ Moderate shortness of breath<br>❑ Severe shortness of breath
One month before your illness with COVID-19, how would you rate your level of loss of taste?	❑ No loss of taste<br>❑ Slight loss of taste<br>❑ Moderate loss of taste<br>❑ Severe loss of taste
When your COVID-19 illness was at its worst how would you rate your level of loss of taste?	❑ No loss of taste<br>❑ Slight loss of taste<br>❑ Moderate loss of taste<br>❑ Severe loss of taste
How would you rate your level of loss of taste?	❑ No loss of taste<br>❑ Slight loss of taste<br>❑ Moderate loss of taste<br>❑ Severe loss of taste
One month before your illness with COVID-19, how would you rate your level of loss of smell?	❑ No loss of smell<br>❑ Slight loss of smell<br>❑ Moderate loss of smell<br>❑ Severe loss of smell
When your COVID-19 illness was at its worst how would you rate your level of loss of smell?	❑ No loss of smell<br>❑ Slight loss of smell<br>❑ Moderate loss of smell<br>❑ Severe loss of smell
How would you rate your level of loss of smell?	❑ No loss of smell<br>❑ Slight loss of smell<br>❑ Moderate loss of smell<br>❑ Severe loss of smell
One month before your illness with COVID-19, how would you rate your level of hair loss?	❑ No hair loss<br>❑ Slight hair loss<br>❑ Moderate hair loss<br>❑ Severe hair loss
When your COVID-19 illness was at its worst how would you rate your level of hair loss?	❑ No hair loss<br>❑ Slight hair loss<br>❑ Moderate hair loss<br>❑ Severe hair loss
How would you rate your level of hair loss?	❑ No hair loss<br>❑ Slight hair loss<br>❑ Moderate hair loss<br>❑ Severe hair loss

Patient Health Questionnaire-4 (PHQ-4)<br>Over the last two weeks, how often have you been bothered by the following problems?	
Feeling nervous, anxious, or on edge	❑ Not at all<br>❑ Several days<br>❑ More than half the days<br>❑ Nearly every day
Not being able to stop or control worrying	❑ Not at all<br>❑ Several days<br>❑ More than half the days<br>❑ Nearly every day
Feeling down, depressed, or hopeless	❑ Not at all<br>❑ Several days<br>❑ More than half the days<br>❑ Nearly every day
Little interest or pleasure in doing things	❑ Not at all<br>❑ Several days<br>❑ More than half the days<br>❑ Nearly every day
**EQ-5D-5L**<br>Please mark the ONE box that best describes your health TODAY.	
Mobility	❑ I have no problems in walking about<br>❑ I have slight problems in walking about<br>❑ I have moderate problems in walking about<br>❑ I have severe problems in walking about<br>❑ I am unable to walk about
Self-Care	❑ I have no problems washing or dressing myself<br>❑ I have slight problems washing or dressing myself<br>❑ I have moderate problems washing or dressing myself<br>❑ I have severe problems washing or dressing myself<br>❑ I am unable to wash or dress myself
Usual activities	❑ I have no problems doing my usual activities<br>❑ I have slight problems doing my usual activities<br>❑ I have moderate problems doing my usual activities<br>❑ I have severe problems doing my usual activities<br>❑ I am unable to do my usual activities
Pain/Discomfort	❑ I have no pain or discomfort<br>❑ I have slight pain or discomfort<br>❑ I have moderate pain or discomfort<br>❑ I have severe pain or discomfort<br>❑ I have extreme pain or discomfort
Anxiety/Depression	❑ I am not anxious or depressed<br>❑ I am slightly anxious or depressed<br>❑ I am moderately anxious or depressed<br>❑ I am severely anxious or depressed<br>❑ I am extremely anxious or depressed

Supplemental Tables

Table S1. Participants were asked to rate the severity of the 8 COVID-19 symptoms shown in this table. The severity of each symptom was classified as: 0 (none), 1 (slight), 2 (moderate), or 3 (severe). At the time of survey completion 6-11 months after the initial positive SARS-CoV-2 test, participants were asked to classify the severity of each symptom for three time points: one month before COVID-19 (pre-illness baseline), at the worst point during acute COVID-19 (peak symptoms), and currently at the time of survey completion. The grading of symptoms for the baseline and peak timepoint relied on the memory of symptoms that were present earlier.

**Symptom**	**None**	**Slight**	**Moderate**	**Severe**
Fatigue	0	1	2	3
Difficulty sleeping	0	1	2	3
Difficulty concentrating	0	1	2	3
Difficulty with completing usual daily tasks	0	1	2	3
Shortness of breath	0	1	2	3
Loss of taste	0	1	2	3
Loss of smell	0	1	2	3
Hair loss	0	1	2	3

Table S2. Covariables used in multivariable regression models.

Variable	Categories
Age	Analyzed as a continuous variable
Race	2 categories: Black; not Black
Ethnicity	2 categories: Hispanic; not Hispanic
Education level	7 categories: Don't know or not answered; Kindergarten or below; Grades 1-8; Grades 9-11; Grade 12 or GED; College 1-3 years; College 4 years or more
Income level	6 categories: Don't know or not answered; Less than $25,000; $25,000-$34,000; $35,000-$49,000; $50,000-$74,000; $75,000 or more
Overall health status	EQ-VAS measured on a continuous scale from 0 to 100

Table S3. The most severe persistent COVID-19 symptom at 6 to 11 months after a positive SARS-CoV-2 test among 176 people with mild acute COVID-19 treated as an outpatient. These 176 people responded to Part 1 of the Twilio survey that they had persistent symptoms at the time of survey completion.

**Most severe COVID-19 symptom at 6–11-month follow-up**	**n (% out of 176 participants)**
Fatigue	55 (31.3%)
Shortness of breath	35 (19.9%)
Difficulty with concentration	16 (9.1%)
Loss of smell	16 (9.1%)
Hair loss	12 (6.8%)
Difficulty sleeping	11 (6.3%)
Difficulty performing usual tasks	9 (5.1%)
Loss of taste	9 (5.1%)
Anxiety	6 (3.4%)
Depression	6 (3.4%)
Missing	1 (0.6%)
